# Analysis of floristic composition and species diversity of vascular plants native to the State of Palestine (West Bank and Gaza Strip)

**DOI:** 10.3897/BDJ.10.e80427

**Published:** 2022-05-19

**Authors:** Mohammed Saleem Ali-Shtayeh, Rana Majed Jamous, Salam Yousef Abuzaitoun

**Affiliations:** 1 Biodiversity and Environmental Research Center, Nablus, Palestine Biodiversity and Environmental Research Center Nablus Palestine

**Keywords:** diversity, endemism, threatened plants, vascular plants

## Abstract

This study aims at providing an updated checklist of the native vascular flora of the Palestinian West Bank and Gaza Strip (State of Palestine, SP), serving as a taxonomic and nomenclatural basis for botanical research and encouraging new floristic surveys and biosystematic studies. The study provides an up-to-date checklist of native vascular taxa of the SP and their distribution within the plant districts in the country. This is the very first annotated checklist for the native vascular plants of the SP which incorporates recent name changes, new distribution records, habitat, herbarium specimens catalouge, Red List criteria, wild edible plants, endemism and use in Traditional Palestinian Herbal Medicine. The quantitative analysis of the flora has shown that the SP hosts 1826 taxa, distributed in 686 genera and 108 families; five taxa are gymnosperms, nine taxa are Pteridophytes and 1812 taxa are angiosperms. The most represented families are Leguminosae (222 taxa, 12.2%), Asteraceae (197, 10.2%) and Poaceae (196, 10.7%), while the most represented genera are *Trifolium* (38, Leguminosae), *Silene* (32, Caryophyllaceae), *Astragalus* (27, Leguminosae), *Medicago* (26, Leguminosae), *Allium* (25, Amaryllidaceae) and *Euphorbia* (25, Euphorbiaceae). Annuals (52.4%), Hemicryptophytes (20.2%) and Chamaephytes (12.2%) are the most represented life-forms amongst the SP flora. The richest plant districts in the SP vascular plant taxa are Gaza Strip (GS) (1216 taxa), Jerusalem and Hebron Mountains (JHM) (1235) and Nablus Mountains (NM) (1126). Agglomerative hierarchical clustering (AHC) permitted the division of SP into two main regions, based on the existence of vascular plant taxa: Region 1 (western plant districts with 1128-1237 taxa) with higher water availability and temperate Mediterranean climate which permit the establishment of more than 65% of the total SP flora in these districts and Region 2 (eastern plant districts with 571-698 taxa), characterised by desert and semi-desert conditions, as well as the presence of alluvial and co-alluvial soils, which allow the survival of lower numbers of plant taxa. One hundred and sixty-five taxa of the SP flora are endemic and near-endemic. However, in comparison with some countries of the Mediterranean Basin, this number is below the average endemism concentration, along with other southern arid countries, such as Tunisia and Egypt. In total, there are 102 threatened plant taxa, belonging to 39 families and 83 genera representing 5.6% of the total plants in the SP. IUCN and the Conservation Measures Partnership (CMP) unified classification of direct threats for SP Red-Listed plants has shown a high extinction risk to the Palestinian threatened wild flora, with 76.5% of the threatened species being either critically endangered (CR) or endangered (EN); only 23.5% were vulnerable (VU). However, several taxa are threatened by numerous factors including small population size, human activities, for example, conversion of traditional to intensive agriculture accompanied by deep ploughing and the application of pesticides, urban development and construction, global climatic change, drying of marshes and wetlands, quarrying, fires and pollution. This checklist can help focus conservation efforts and provide a framework for research, protection and policy applications for the SP flora, especially for the endemic and threatened plants.

## Introduction

The flora of the State of Palestine, SP (West Bank and Gaza Strip) has been partly covered by several authorities including Flora Palaestina (Post 1932, Post 1933, Zohary 1966, Zohary 1972, Danin 1988, Feinbrun-Dothan and Danin 1991, Danin 1992, Sapir et al. 2003, Danin 2004, Shmida and Pollak 2007, Horvitz and Danin 2015).

Far fewer studies have dealt with the flora of the SP (Ali-Shtayeh and Jamous 2018). The latter study provided a preliminary checklist of vascular plants of the West Bank and Gaza Strip (State of Palestine, SP). The checklist has shown that the vascular flora of the SP comprises 1938 taxa in 733 genera and 111 families. The checklist, however, presented few taxonomic, morphological, chorological and ecological data on each plant in the list, including life form, chorotype and pollination type. It also included 79 introduced taxa, 18 extinct, 10 with mistaken distribution and eight synonyms. In addition, no herbarium specimens were cited for the taxa included, nor was a quantitative floristic analysis provided.

On the other hand, the Red-Listing of threatened vascular plant taxa has received special attention (Ali-Shtayeh and Jamous 2002, Ali-Shtayeh and Jamous 2018). Such a Red List can help focus conservation efforts and provide a framework for research, conservation and policy applications for the SP flora, especially the endemic and threatened plants. The 2018 Plant Red list of the SP flora comprises 102 taxa and suggested conservation recommendations for threatened plants. The threatened vascular plant species (102 taxa) listed in the Red List were assessed on the basis of taxonomy, distribution, population size and trends, habitats and ecology and threats, in accordance with IUCN Red List Categories and Criteria, Version 3.1. The “red number” method (Sapir et al. 2003) with some modifications (Ali-Shtayeh and Jamous 2018) was used, as complementary to IUCN Criteria, to calculate the red numbers for the plants in order to identify the threshold for the endangered status of a plant species and rank the degree of risk. The modified “red-number” method uses six quantitative criteria (rarity, habitat vulnerability, attractiveness, distribution type (endemism), disjunctiveness and peripherality) which are combined into a single quantitative index known as the “red number”. Each criterion has its own scale, which reflects its relative weight in determining the extent to which a certain plant species is endangered.

A couple of local studies “reported” a preliminary checklist of 1612 plants of the West Bank (Al-Sheikh et al. 2000, Al-Sheikh 2019, Al-Sheikh and Qumsiyeh 2021) and a third one published a preliminary checklist of 600 rare plants in the area (Al-Sheikh and Qumsiyeh 2021). However, the three studies were found to be flawed and all share the following characteristics: (a) the methods used to identify species are either not reported or are inappropriate, (b) the study methods are not described in sufficient detail or clarity to enable the reader to repeat them, (c) the statistical methods used in the research are either not suitable and/or the assumptions on which they are based do not fit the data that were used to analyse and (d) the results and conclusions are unclear, incorrect, meaningless or incomprehensible (Altman and Moher 2014,Buxton et al. 2021, Ford et al. 2021, Peres et al. 2021).

In the present study, we examine plant diversity in the SP and explore the potential influence of variation in plant districts and habitats on such diversity. We undertook the IUCN-CMP Unified Classification of Direct Threats (Version 3.2) and Conservation Actions Needed (Version 2.0) to the threatened plant species affected by the first level of threat types in order to determine the main causes threatening the existence of the plant and conservation priorities or human-activity-related extinction probability.

Our aims were: (1) to update the checklist of the SP native vascular plants flora. The update combines published and unpublished lists and survey reports to include all names that have been used and are in use for the flora of SP, with additional information on taxonomy, morphology, ecology and herbarium specimens cited for the taxa included; (2) to describe species distributions over the whole region and locate areas of highest interest for plant diversity; (3) to evaluate the influence of plant district, location and habitat on the floristic diversity of sites; (4) to identify the main causes threatening the existence of the plant and conservation priorities or human-activity-related extinction probability.

## Materials and methods

### Study area and current vegetation status

The State of Palestine (West Bank and Gaza) (SP) is located within historic Palestine, with a total area of 6257 km^2^. The geographical location of SP is between 31°13’ and 32°33’ Latitude and between 34°13’ and 35°34’ Longitude. In spite of its small area, SP is categorised by a pronounced variation in topography and climate. Five topographical zones can be distinguished in SP, including the Central Highlands, Semi-Coastal Region, Eastern Slopes, Jordan Rift Valley and Coastal Region (Ali-shtayeh et al. 2003). The SP can be divided into two climatic regions: the Eu-Mediterranean climate, which includes the coastal plain, the mountain range and the central hills. It is characterised by a mild rainy winter that lasts about six months and a warm, dry summer, with a rainfall rate ranging between 350-1000 mm; and the Xero-tropical climate zone, which includes the Jordan Valley, is characterised by warm winter and extremely hot dry summer and a lack of rainfall. These conditions are considered insufficient to support continuous plant life (Shtayeh and Hamad 1995).

The average annual temperature in the Mediterranean climate zone ranges between 17 and19°C and its average in the hottest months of August is between 22 and26°C and in the coldest months of January between 8 and10°C. The temperature may rise to 40°C in summer and fall to -6°C in winter. In the Jordan Valley, the average annual temperature ranges between 21 and25°C and its average in the hottest months (August) ranges between 21 and32°C and in the coldest months of January between 4 and6°C. The temperature may reach 48°C in summer and drop to -2°C in winter.

The SP can also be divided into four phytogeographical regions including the Mediterranean, the Irano-Turanian, the Saharo-Arabian and the Sudanese Penetration regions (Zohary 1972, Fienburn-Dothan 1978, Fienburn-Dothan 1986). Despite the small area of the SP, this confluence of the four regions has led to the rich diversity in plant communities and their components.

**Figure 1.**
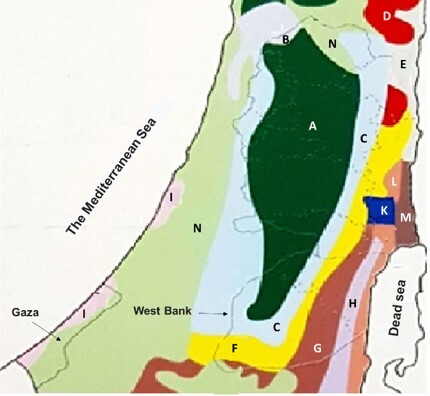
Vegetation map of the Palestinian West Bank and Gaza Strip. A, Maquis and forests; B, Park forest of *Quercusithaburensis*; C, Park forest of *Ceratoniasiliqua* and *Pistacialentiscus*; D, *Ziziphuslotus* with herbaceous vegetation; E, Savannoid Mediterranean vegetation;F, Semi-steppe batha; G, Steppe vegetation; H, Desert vegetation; I, Sand vegetation; K, Oases with Sudanian trees; L, Desert savannoid vegetation, with swamps and reed thickets; M, Wet salines; N, Synanthropic vegetation: with *Ziziphusspina-christi* (L.) Desf., and *Acaciaraddiana* trees.

**Figure 2.**
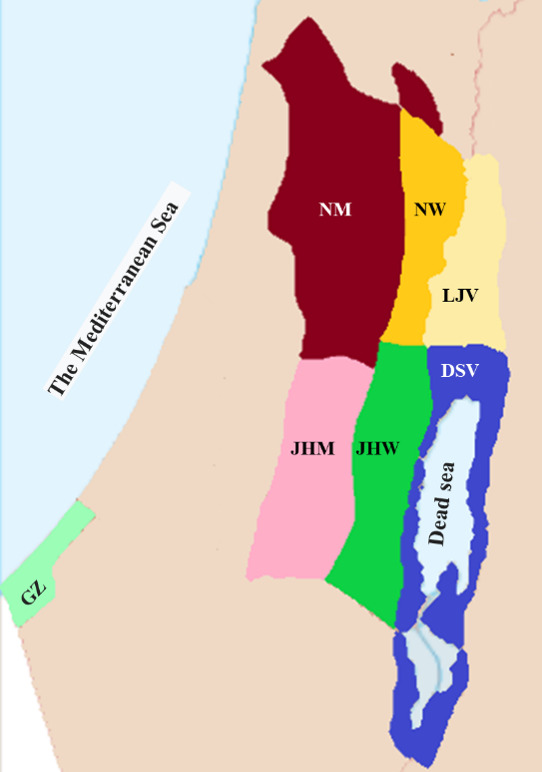
Plant geographical districts: NM, Nablus Mountains; NW, Nablus Wilderness; LJV, Lower Jordan Valley; JHM, Jerusalem and Hebron Mountains; JHW, Jerusalem and Hebron Wilderness; DSV, Dead Sea Valley; and Gaza Strip (GS).

The vegetation of SP comprises 13 vegetation types (Danin 1988, Shtayeh and Hamad 1995) (Fig. 1 Table 1) . The area of the SP also comprises seven plant geographic districts: Nablus Mountains (NM), Nablus Wilderness (NW), Lower Jordan Valley (LJV), Jerusalem and Hebron Mountains (JHM), Jerusalem and Hebron Wilderness (JHW), Dead Sea Valley (DSV) and Gaza Strip (GS) (Zohary 1966) (Fig. 2Table 1). NM and JHM plant districts include also a narrow strip of the Jerusalem and Hebron Mountains foothills. The distribution of vegetation types in the plant districts of the SP is presented in Table 1.

### Specimen collection and identification

Between November 2011 and May 2020, the research team consisting of botanists from the Biodiversity and Environmental Research Center (BERC) carried out several explorations (surveys) in the SP. A combination of standard sampling methods for plant specimen collection and sight observation was used to aid the characterisation of the vegetation (Danin 1988, Al-Eisawi and Oran 2015). Specimen details included life form, habitat, distribution and collector details. The specimens were then preserved by pressing. Voucher specimens were deposited at BERC Herbarium, Til, Nablus, SP, labelled with the plant’s scientific name and common names, collection date, GPS coordinates, habitat, collector, identifier and a herbarium specimen number.

The current study was also based on reviews of numerous publications and databases. These resources include previous Floras and available literature (Post 1932, Post 1933, Zohary 1966, Zohary 1972, Fienburn-Dothan 1978, Fienburn-Dothan 1986, Govaerts 1995, Govaerts 1996, Al-Eisawi 1998, Govaerts 1999, Plitmann et al. 1999, Al-Eisawi 2013, Oran 2014, Ali-Shtayeh and Jamous 2018 and an exhaustive survey of Web of Sciences, Scopus and Google Scholar, to check for new taxa descriptions and/or taxonomic revisions. Local herbaria (e.g. BERC Herbarium) and online global databases were consulted (see Table 2 for details).

The current checklist is an additional update, combining published and unpublished lists and survey reports to include all names that have been used and are in use for the flora of SP, with additional information on taxonomy, morphology, ecology and herbarium specimens cited for the taxa included. However, the taxa which are introduced (79), extinct (18), with mistaken distribution (10) and synonyms (8) are removed from the 2018 checklist.

Supplementary to the Palestinian plant checklist (Ali-Shtayeh and Jamous 2018), the additional data include life form, habitat, distribution, plant geographical districts, abundance, herbarium specimens’ catalogue, Red List Criteria, wild edible plants, traditional Palestinian medicinal plants and endemism (Ali-Shtayeh and Jamous 2008, Ali-Shtayeh et al. 2008, Ali-Shtayeh et al. 2014, Ali-Shtayeh and Jamous 2018). The nomenclature of plant taxa follows World Flora Online (WFO 2021) (previously The Plant List, TPL) and the International Plant Names Index (IPNI 2017) databases. Genera and species were reported in families as recognised by the Angiosperm Phylogeny Group IV (APG IV 2016). Life forms were identified following the system of Raunkiaer (1977). For families of monocotyledonous plants, accepted names and synonymy are as given in eMonocot (http://e–monocot.org/).

The distribution and habitat of the plant taxa amongst the administrative districts of the Palestinian West Bank and Gaza Strip were determined according to locations reported on herbarium specimen labels and in literature. The distribution data for the vascular plants were also coded using the seven plant geographical districts of the SP: NM, NW, LJV, DSV, JHW, JHM and GS. The symbols for the distribution of plants in floristic districts are VC (very common), C (common), R (rare) and VR (very rare).

In the chorology (chorotype) column (Ch) of the floristic checklist, the chorological categories for native vascular plants taxa were coded as outlined in Ali-Shtayeh and Jamous (2018): M, Mediterranean; IT, Irano-Turanian; SA, Saharo-Arabian; ES, Euro-Siberian; COSM, Cosmopolitan; SUD, Sudanian; and T, Tropical.

In the life-form column (Lf) of the floristic checklist, the life-form categories for the native vascular plants of the Palestinian flora were coded following Raunkiaer (1934) and subsequent extensions by Ellenberg and Mueller-Dombois (1965): A, Annuals; F, Biennials; C, Sub-shrubs and Chamaephytes; G, Geophytes; H, Hemicryptophytes; P, Parasites; Q, Aquatic plants; S, Shrubs; PhS, Phanerophyte shrub; T, Trees; V, Vines; and HE, Helophyte.

### Data analysis

A data matrix of 1826 taxa and seven plant geographical districts was developed and agglomerative hierarchical clustering (AHC) was carried out using SPSS V21 to construct homogeneous classes of floristic districts on the basis of their Jaccard dissimilarity in the composition of plant taxa (Roleček et al. 2009).

Analysis of floristic composition and species diversity of threatened plants (102 species) and the classification of direct threats to these species were based on the updated 2018 Red Plant List of the SP (Ali-Shtayeh and Jamous 2018). In the latter study, the threatened vascular plant species were assessed on the basis of taxonomy, distribution, population size and trends, habitats and ecology and threats, in accordance with IUCN Red List Categories and Criteria, Version 3.1. The “red number” method (Sapir et al. 2003) with some modifications was used, as complementary to IUCN Criteria, to calculate the red numbers for the plants in order to identify the threshold for the endangered status of a plant species and rank the degree of risk. The modified “red-number” method uses six quantitative criteria including rarity, habitat vulnerability, attractiveness and distribution type (endemism), which are combined into a single quantitative index known as the “red number”. Each criterion has its own scale, which reflects its relative weight in determining the extent to which a certain plant species is endangered.

### Unified Classifications of Threats and Conservation Actions

To determine the main causes threatening the existence of the plant and conservation priorities or human-activity-related extinction probability, we applied the IUCN – Conservation Measures Partnership (IUCN-CMP) Unified Classification of Direct Threats (Version 3.2) (Table 8) and Conservation Actions Needed (Version 2.0) to the percentage of the 102 threatened plant species affected by the first level of threat types (156 total threats; threat data were from Ali-Shtayeh and Jamous (2018)) (IUCN 2019); the results are presented in Table 8 and Fig. 12. The application of these classifications offers an indication as to the main causes threatening the existence of the plants. It also helps determine conservation priorities or human-activity-related extinction probability and is expected to help practitioners more systematically identify threats and appropriate actions.

## Results and discussion

### Checklist

A comprehensive floristic checklist of the vascular plant taxa of the SP area is presented in Suppl. material 1. Within this checklist, species are organised alphabetically in each family. The families are divided into three groups, namely pteridophytes, gymnosperms and angiosperms. Families of pteridophytes are organised, based on the PPG I system (PPG I 2016), while those of gymnosperms are organised, based on Christenhusz et al. (2011) and families of angiosperms are organised based, on the APG IV system (APG IV 2016).

According to our results, SP hosts 1826 plant taxa. This is in partial disagreement with our previous checklist (Ali-Shtayeh and Jamous (2018); 1938 taxa). The reduced number of total flora in SP is mainly due to the exclusion of introduced (79) and extinct taxa (18) from the current checklist. Additionally, five taxa were excluded because they were synonymised with other taxa. In addition, 10 taxa were excluded due to recent updates in their geographical distribution.

As this checklist is also present in an electronic format, it is relatively easy to keep the plants of SP up to date and we hope that it will form the basis of a national flora of SP.

### Diversity within the native vascular flora of the Palestinian West Bank and Gaza Strip

The native vascular flora of the SP comprises 1826 taxa in 686 genera belonging to 108 families of flowering plants (Suppl. material 1). The Pteridophytes are represented by three families, six genera and nine taxa. The Gymnosperms are represented by three families, three genera and four taxa. The angiosperms consist of 1813 species grouped in 102 families and 677 genera. The top 10 species-rich families are shown in (Table 3). The families Leguminosae (222 spp, 12.1%), Compositae (Asteraceae) (197 spp, 10.8%), Poaceae (196 spp, 10.7%), Brassicaceae (85 spp, 4.6%), Caryophyllaceae (84 spp, 4.6%) and Lamiaceae (80 spp, 4.4%) are the largest with more than 80 species each. *Trifolium* (38 spp, Leguminosae), *Silene* (32 spp, Caryophyllaceae), *Astragalus* (27 spp, Leguminosae), *Medicago* (26 spp, Leguminosae), *Allium* (25 spp, Amaryllidaceae) and *Euphorbia* (25 spp, Euphorbiaceae) are the largest genera with more than 20 species in each (Table 4).

### Distribution of Palestinian flora

**Figure 3.**
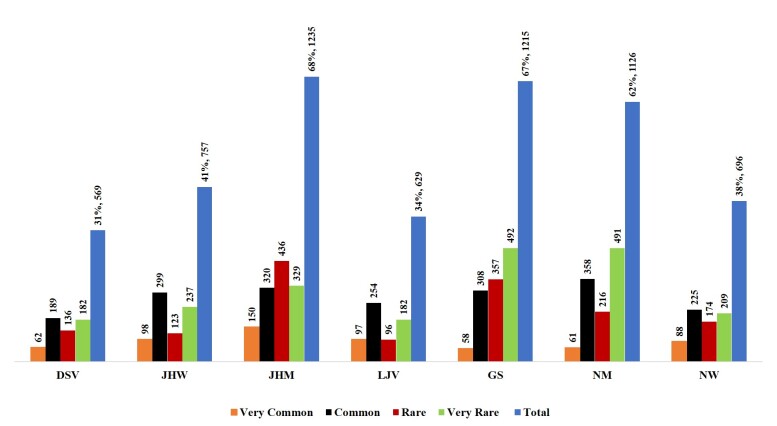
The plant species richness distributed across the plant districts. DSV: Dead-Sea Valley, JHW: Jerusalem & Hebron Wilderness, JHM: Jerusalem & Hebron Mountains, LJV: Lower Jordan Valley, GS: Gaza Strip, NM: Nablus Mountains, NW: Nablus Wilderness. C: Common, VC: Very common, R: Rare, VR: Very Rare.

The plant species richness in absolute numbers is not uniformly distributed across the plant districts; as a general pattern, GS (1216 taxa), JHM (1235) and NM (1127) are richer in absolute numbers of native vascular plants than other districts (Fig. 3).

**Figure 4.**
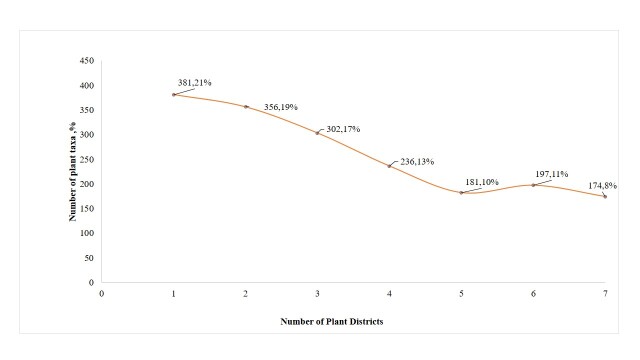
Occurrence of Palestinian plant taxa across the different plant districts.

Out of the 1826 plant taxa present in SP 173 taxa (10%) occur in all plant districts, 380 taxa (21%) are plants whose occurrence is confined to a single district, 356 (19%) taxa are recorded in two, 302 (17%) taxa in three, 236 (13%) taxa in four and 181 (10%) in five plant districts (Fig. 4).

**Figure 5.**
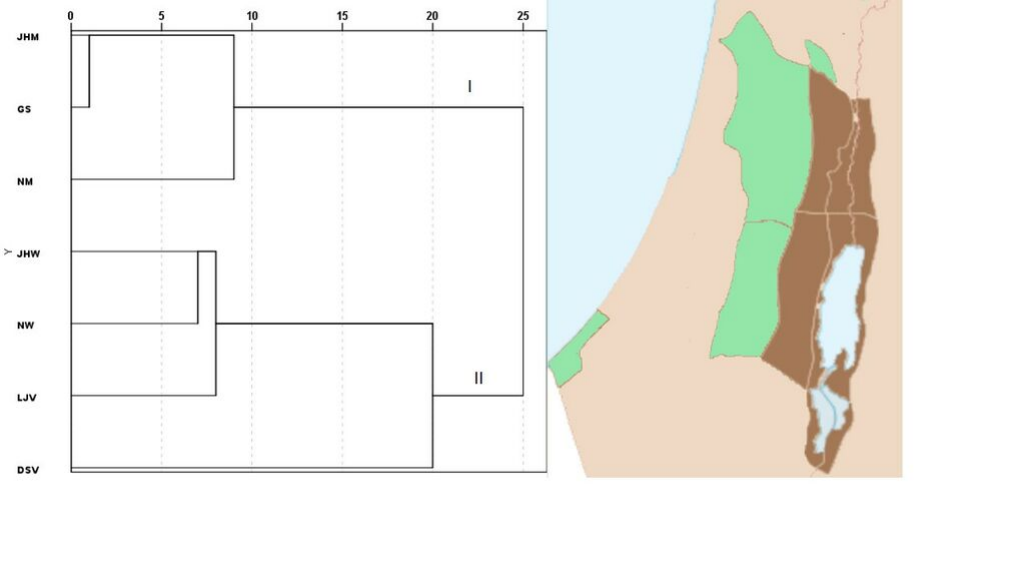
Agglomerative hierarchical cluster (AHC) dividing the plant districts regions in SP into two groups: I (western districts), II (eastern districts). DSV: Dead-Sea Valley, JHW: Jerusalem & Hebron Wilderness, JHM: Jerusalem & Hebron Mountains, LJV: Lower Jordan Valley, GS: Gaza Strip, NM: Nablus Mountains, NW: Nablus Wilderness.

The AHC analysis divided the SP into two principal regions according to the presence of plants; cluster 1 includes the western plant districts JHM, NM and GS characterised by high rainfall as well as mountainous, hilly, semi-coastal and coastal topography with mainly Terra rossa and alluvial soils; and cluster 2 includes the eastern plant districts JHW, NW, DSV and LJV characterised by desert and semi-desert conditions, as well as the alluvial and coalluvial soils (Fig. 5). The limited availability of water in arid and semi-arid eastern plant districts (cluster 2) allows the survival of lower numbers of plant taxa (571-698 taxa) compared with the western plant districts (1128-1237 taxa, cluster 1) with higher water availability and temperate Mediterranean climate which permit the establishment of more than 65% of the total SP flora in these districts.

### Habitat preferences of Palestinian plant taxa

Our evaluation of the habitat preferences of plant taxa (Table 5) in the SP has revealed that the most common are plants of batha habitat (41.5%), followed by plants of the desert (15.9%) and humid (11.6%) habitats. On the other hand, the least common are plants of walls (0.1%), Mediterranean grasslands (0.3%) and shady rocks (0.5%) habitats.

### Distribution of life forms in the Palestinian flora

Most of the plants in the Palestinian flora are annuals (52.5%), followed by hemicryptophytes (20.2%) and chamaephytes (12.2%). Herbaceous plants (annual, geophytes and hemicryptophytes) constitute most of the flora (81.8%), followed by woody plants (chamaephytes, phanerophyte shrubs and trees) (18.2%) (Table 6).

### Chorotype

Species in the plant list are categorised on the basis of their distribution type (chorotype) (Table 7). Each plant is grouped according to its main chorotype. About 59.9% of the species in the plant list are Mediterranean, 12.0% are Irano-Turanian and 11.8% are Saharo-Arabian.

### Climatic regions

**Figure 6.**
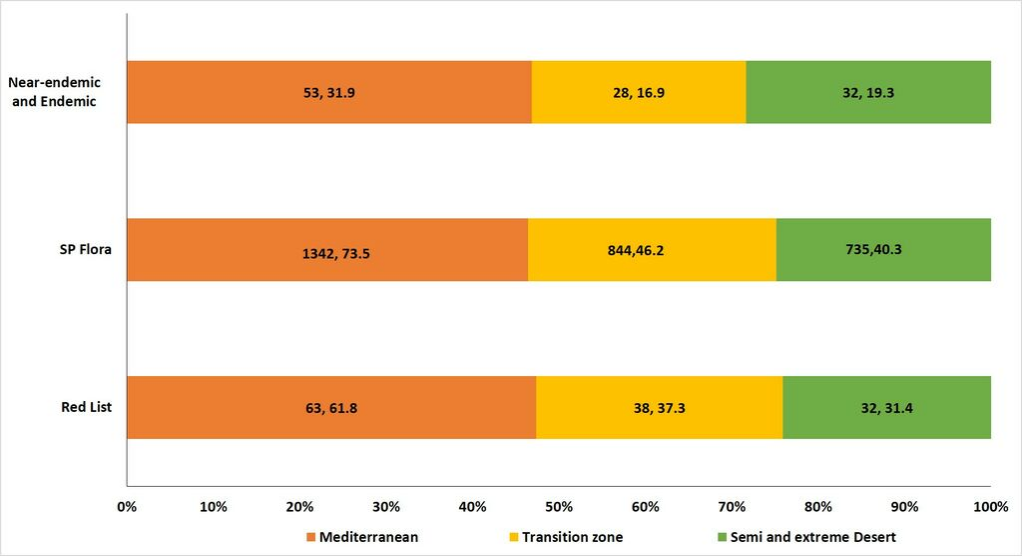
Distribution of the Palestinian flora on the different climatic zone.

An analysis of the distribution of the flora in the different ecogeographic regions shows the Mediterranean climatic region of the SP to be the most species-rich (1342 spp, 73.5%), followed by the semi- and extreme desert (844 spp, 46%) and transition zone climatic regions (735 spp, 40.3%) (Fig. 6).

### Pollination system

The main pollination system in the Palestinian flora is “Animals”. Of the plant list, 1424 (78.0%) of the species are animal pollinated, while 378 (20.7%) are wind-pollinated.

### Endemic plant diversity within the vascular flora of SP

**Figure 7.**
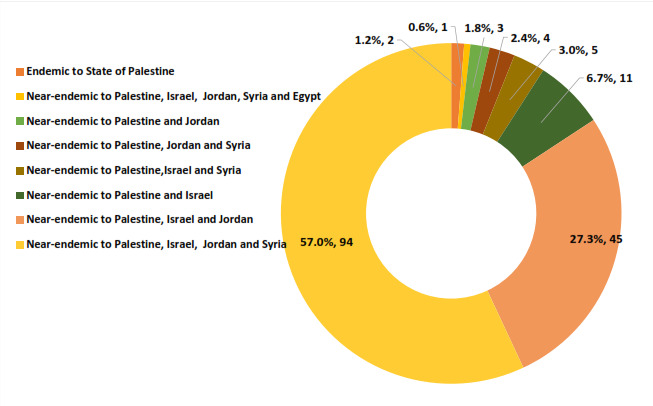
Near-endemic and endemic plant diversity within the vascular flora of the State of Palestine.

One hundred and sixty-five plant species (9.0% of the total Palestinian flora) are near-endemic to the SP and one or more of the neighbouring countries (Israel, Jordan, Syria, Lebanon and Egypt). Two taxa (*Ferulasamariae* Zohary & P. H. Davis and Irislortetiivar.samariae (Dinsm.) Feinbrun) are endemic only to SP. Ninety-four spp (56.6%) are endemic to SP, Israel, Jordan and Syria; 45 spp (27.1%) are endemic to SP, Israel and Jordan; 12 spp (7.2%) are endemic to SP and Israel; five spp are endemic to SP, Israel and Syria (3.0%); four spp are endemic to SP, Jordan and Syria (2.4%); and three spp (1.8%) are endemic to SP and Jordan and one species (0.6%) is endemic to SP, Israel, Jordan, Syria and Egypt (Fig. 7). However, in comparison with some countries of the Mediterranean Basin, this number is below the average endemism concentration, along with other southern arid countries, such as Tunisia and Egypt (Rankou et al. 2013, Fois et al. 2017, Abdeaal et al. 2018).

### Distribution of Palestinian near-endemic flora

All plant districts in SP harbour at least one near-endemic taxon, the most near-endemic rich region is NM with 102 near-endemic taxa; followed by JHM and GS with 99 and 88 near-endemic taxa, respectively (Fig. 9).

Nine endemic taxa occur in all of the SP plant districts, forty-three taxa are endemics whose occurrence is confined to just a single region and forty endemic taxa are recorded only in two plant districts (Fig. 10).

The most widespread endemic taxa were: *Vagariaparviflora* (Desf. ex Delile) Herb, *Chaetosciadiumtrichospermum* (L.) Boiss., *Echiumjudaeum* Lacaita, *Campanulahierosolymitana* Boiss., *Campanulastellaris* Boiss., *Galiumjudaicum* Boiss., *Solanumsinaicum* Boiss, *Silenegrisea* Boiss. and *Verbascumeremobium* Murb.

### Habitat preferences of Palestinian near-endemic plant taxa

Our evaluation on the habitat preferences of endemic plant taxa (Table 5) in the State of Palestine has revealed that the most common are endemic plants of batha habitat (45%), followed by plants of sand with 18.7%, desert and shrub steppes (12.7%). On the other hand, the least common are near-endemic plants of Mediterranean strand, ruderal and nutrient-rich soils (1%).

### Distribution of life forms in the Palestinian near-endemic plant taxa

Similar to that in the Palestinian flora, the majority of endemic plant taxa are mainly annuals at 50%, followed by hemicryptophytes at 21.8% (Table 6). Herbaceous plants constitute most of the endemic flora (90.4%), followed by woody plants (16.3%).

### Threatened plants

In total, there are 102 threatened plant taxa (Ali-Shtayeh and Jamous 2018), belonging to 39 families and 83 genera representing 5.6% of the total plants in the SP.

This study also shows a high extinction risk to the Palestinian threatened wild flora, with 76.5% of the threatened species being either critically endangered (CR) (39.2%) or endangered (EN) (37.3%); only 23.5% were vulnerable (VU) (Fig. 11).

The application of the IUCN-CMP Unified Classification of Direct Threats (Version 3.2) to the percentage of 102 threatened plant species affected by the first level of threat types (156 total threats; threat data were from Ali-Shtayeh and Jamous (2018) (IUCN 2019), has offered an indication as to the main causes threatening the existence of the plants (Table 8 and Fig. 12). These include: Small population size, human activities, for example, conversion of traditional to intensive agriculture accompanied by deep ploughing and the application of pesticides, overexploitation (e.g. overgrazing), urban development and construction, global climatic change, drying of marshes and wetlands, quarrying, fires and pollution (Fig. 12). The application of these classifications offers an indication as to the main causes threatening the existence of the plants. It also helps determine conservation priorities or human-activity-related extinction probability and is expected to help practitioners more systematically identify threats and appropriate actions.

### Distribution of SP threatened plants

All plant districts in the SP harbour at least fifteen of the threatened taxa. The richest areas with threatened plants include JHM with 51 threatened taxa; followed by NM and GS with 44 and 33 threatened taxa, respectively (Fig. 13).

**Figure 8.**
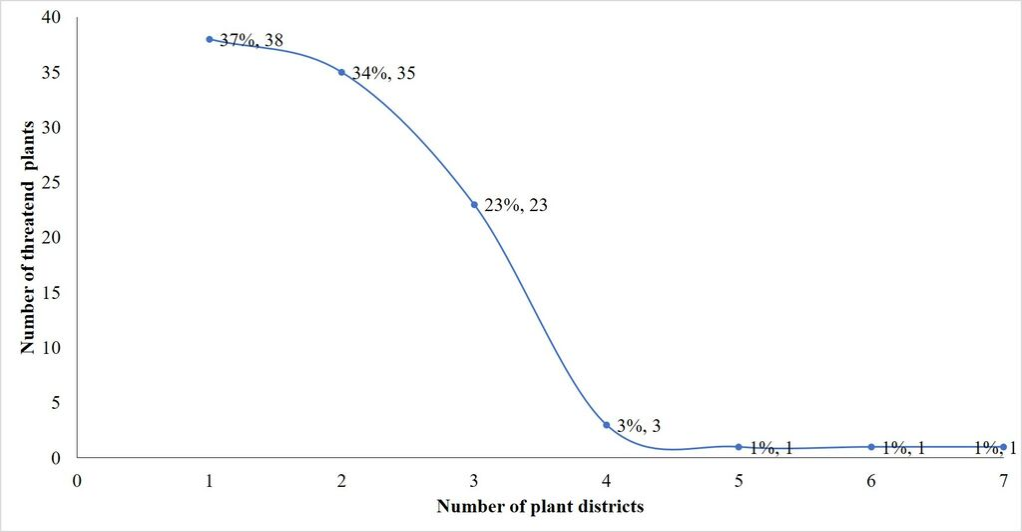
Distribution of SP threatened plants across plant districts.

**Figure 9.**
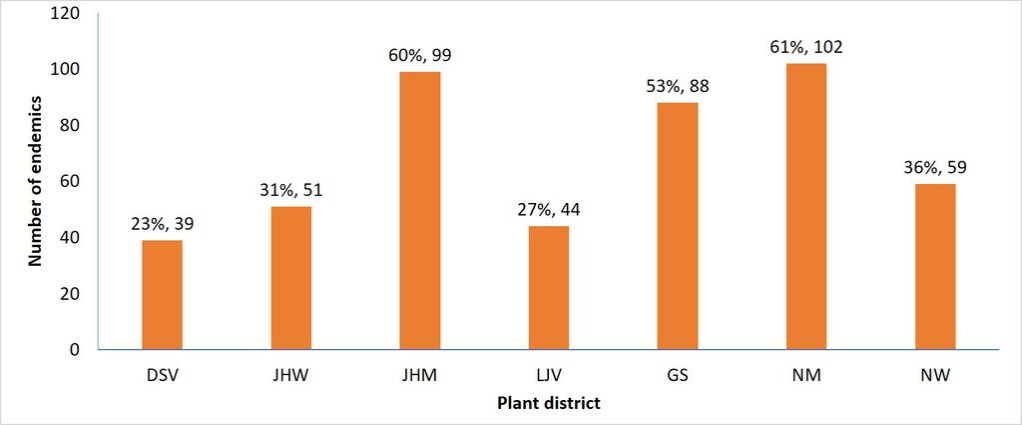
Distribution of the SP near-endemic and endemic flora in various plant districts. DSV: Dead-Sea Valley, JHW: Jerusalem & Hebron Wilderness, JHM: Jerusalem & Hebron Mountains, LJV: Lower Jordan Valley, GS: Gaza Strip, NM: Nablus Mountains, NW: Nablus Wilderness.

**Figure 10.**
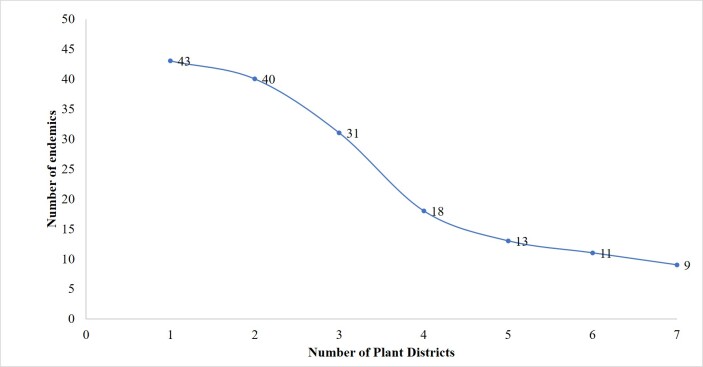
Distribution of near-endemic and endemic flora across plant districts.

**Figure 11.**
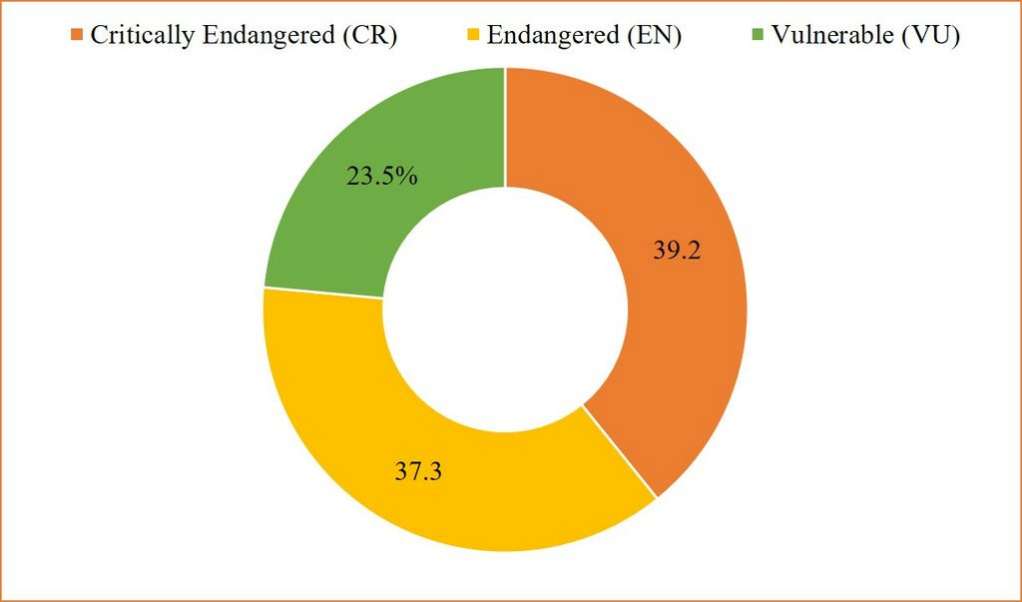
Distribution of threatened categories in the red plants of the State of Palestine.

**Figure 12.**
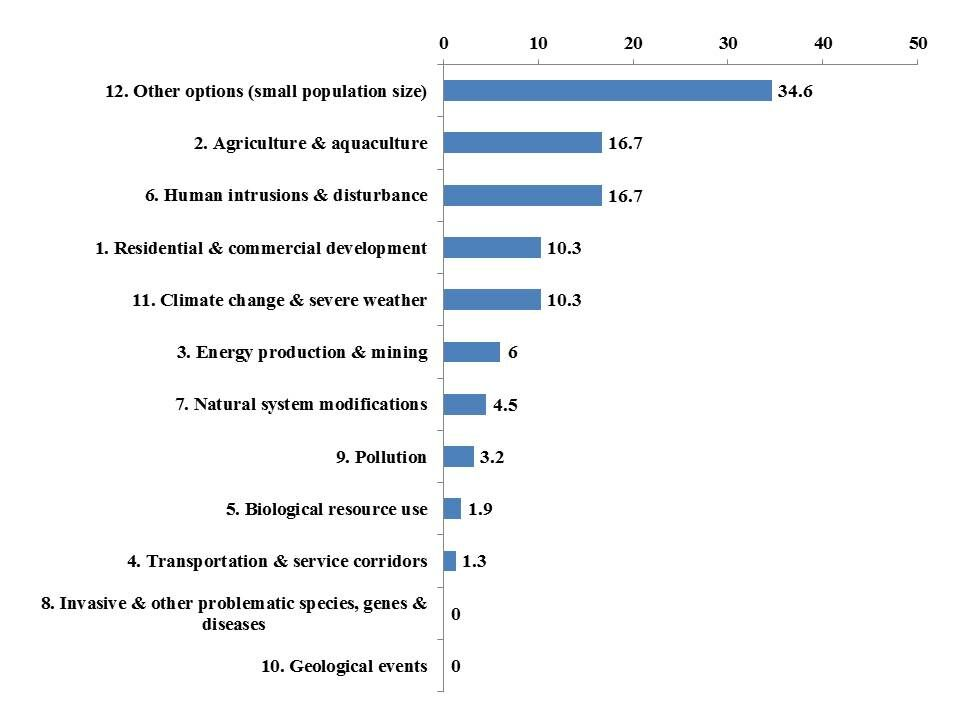
Application of the threat classification to the percentage of 102 threatened vascular plant species affected by the first level of threat types (156 total threats), showing the potential main causes threatening the existence of the plants.

**Figure 13.**
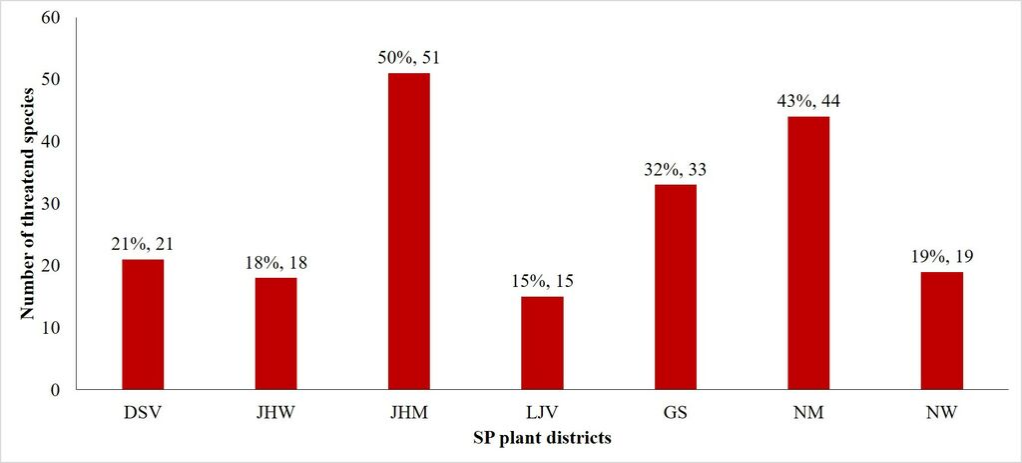
Distribution of the State of Palestine threatened plant species in the different plant districts.

One threatened taxon occurs in all, six or five of the SP plant districts (Fig. 8). On the other hand, thirty-eight threatened taxa are confined to just a single region and thirty-five taxa were recorded in only two plant districts.

The most widespread threatened taxa are *Euphorbiapeplus* L., *Teucriumparviflorum* Schreb. and *Silenepapillosa* Boiss., which occur in at least five SP plant districts.

### Habitat preferences of SP threatened plant taxa

Our evaluation on the habitat preferences of endemic plant taxa in the SP has revealed that the most common are threatened plants of batha habitat (44.1%), followed by plants of the desert with 20.6%, desert, humid habitats (11.8%) and shrub steppes and salty habitats (7.8%) (Table 5). On the other hand, the least common threatened plants are those of the Mediterranean maquis and forest and shady rocks habitats (1%).

### Distribution of life forms in the threatened SP flora

Comparable to that in the Palestinian flora, most of the threatened plants in the Palestinian flora are annuals at 39.2%, followed by hemicryptophytes at 25.5% (Table 6) and are mainly herbaceous (48%) followed by woody plants (41%).

### Chorotype (distribution type) of threatened plants

Threatened plants are categorised on the basis of their distribution type (chorotype). A total of 48.0% of the species in the Red List are Mediterranean species (Table 7), but this proportion is less than that expected according to the Mediterranean chorotype proportion in the SP flora (59.9%). The Irano-Turanin species (27.5%), which grow mainly in oases along the Dead Sea Valley are also over-represented in the list (12.0%), may be due to the presence of water springs along the rift-valley, where those species grow.

## Supplementary Material

054CDAA9-3C8B-5E8A-B7A6-E61F9D84FE95 10.3897/BDJ.10.e80427.suppl1 Supplementary material 1 An updated annotated list of vascular plants native to State of Palestine
Data type list of vascular plants native to State of Palestine File: oo_631708.xlsx https://binary.pensoft.net/file/631708 Mohammed Saleem Ali-Shtayeh, Rana Majed Jamous, Salam Yousef Abuzaitoun

## Figures and Tables

**Table 1.** Distribution of vegetation types in the plant districts of the Palestinian West Bank and Gaza Strip; NM, Nablus Mountains; NW, Nablus Wilderness; LJV, Lower Jordan Valley; JHM, Jerusalem and Hebron Mountains; JHW, Jerusalem and Hebron Wilderness; DSV, Dead Sea Valley; and Gaza Strip (GS).

Unit	Vegetation type	Plant geographical districts						
		GS	NM	NW	LJV	DSV	JHW	JHM
A	Maquis and forests		Ö					Ö
B	Park forest of *Quercusithaburensis*		Ö					
C	Park forest of *Ceratoniasiliqua* and *Pistacialentiscus*		Ö	Ö				Ö
D	*Ziziphuslotus* with herbaceous vegetation			Ö				
E	Savannoid Mediterranean vegetation				Ö			
F	Semi-steppe batha			Ö	Ö		Ö	Ö
G	Steppe vegetation						Ö	
H	Desert vegetation						Ö	
I	Sand vegetation	Ö						
K	Oases with Sudanian trees				Ö	Ö		
L	Desert savannoid vegetation; with swamps and reed thickets					Ö		
M	Wet salines				Ö	Ö		
N	Synanthropic vegetation: with *Ziziphusspina-christi* and *Acaciaraddiana* trees	Ö	Ö					
	Vegetation units /district*	I+N	A+B+C	C+D+F	E+F+K+M	K+L+M	F+G+H	A+C+F

* As in Figure 1

**Table 2.** Global databases used for compiling the checklist of natural plants in the State of Palestine (SP).

Database	Link
Global Biodiversity Information Facility (GBIF)	http://www.gbif.org/occurrence
International Plant Names Index (IPNI)	http://www.ipni.org
JSTOR Global Plants	http://plants.jstor.org
Kew World Checklist of Selected Plant Families (WCSP)	http://wcsp.science.kew.org/home.do
Plants of the World Online (POWO)	http://www.plantsoftheworldonline.org
World Flora Online (WFO) (previously The Plant List (TPL))	http://www.worldfloraonline.org (http://www.theplantlist.org)
Lebanon Flora	http://www.lebanon-flora.org/contact.htm
Flora of Israel online	https://flora.org.il/en/plants/
World checklist of selected plant families (WCSP)	http://apps.kew.org/wcsp/
Angiosperm Phylogeny Website	www.mobot.org/mobot/research/apweb

**Table 3.** Plant families with the highest representations of the Palestinian flora.

Families	Number of genera	Number of species	%
Leguminosae	44	222	12.2
Compositae	83	197	10.8
Poaceae	87	196	10.7
Brassicaceae	52	85	4.7
Caryophyllaceae	25	84	4.6
Lamiaceae	25	80	4.4
Apiaceae	44	77	4.2
Amaranthaceae	19	68	3.7
Boraginaceae	21	51	2.8
Plantaginaceae	11	47	2.6

**Table 4.** Plant genera with the highest representations of the Palestinian flora.

Genera	Family	No. of species
*Trifolium*	Leguminosae	38
*Silene*	Caryophyllaceae	32
*Astragalus*	Leguminosae	27
*Medicago*	Leguminosae	26
*Allium*	Amaryllidaceae	25
*Euphorbia*	Euphorbiaceae	25
*Erodium*	Geraniaceae	17
*Salvia*	Lamiaceae	17
*Bromus*	Poaceae	16
*Convolvulus*	Convolvulaceae	15

**Table 5.** Habitat preferences of Palestinian plant taxa.

Habitat	Habitat	The plant list n = 1826	Endemic plants n = 165	Red list n = 102
Batha	A	758 (41.5%)	75 (45.2%)	45 (44.1%)
Desert	B	290 (15.9%)	21 (12.7%)	21 (20.6%)
Humid habitats	C	211 (11.6%)	5 (3%)	12 (11.8%)
Sand	D	159 (8.7%)	31 (18.7%)	7 (6.9%)
Disturbed habitats	E	104 (5.7%)	3 (1.8%)	3 (2.9%)
Mediterranean maquis and forest	F	118 (6.5%)	11 (6.6%)	1 (1%)
Shrub-steppes	G	275 (15.1%)	21 (12.7%)	8 (7.8%)
Hard rock outcrops	H	107 (5.9%)	17 (10.2%)	5 (4.9%)
Cultivated areas (weeds)	I	61 (3.3%)	2 (1.2%)	3 (2.9%)
Salty habitats	J	75 (4.1%)	3 (1.8%)	8 (7.8%)
Nutrient-rich soils	K	34 (1.9%)	1 (0.6%)	0 (0%)
Ruderal	L	34 (1.9%)	1 (0.6%)	0 (0%)
Mediterranean strand	M	27 (1.5%)	1 (0.6%)	3 (2.9)
Tragacanth shrub vegetation (Oro-Mediterranean)	N	10 (0.5%)	0 (0%)	2 (2)
Shady rocks	O	9 (0.5%)	0 (0%)	1 (1%)
Mediterranean grasslands	P	6 (0.3%)	0 (0%)	0 (0%)
Walls	Q	2 (0.1%)	0 (0%)	0 (0%)

**Table 6.** Distribution of life forms in the Palestinian flora.

Life form		Plant list (n = 1826)	Endemic plants (n = 165)	Threatened plants (n = 102)
Annuals	A	959 (52.5%)	83 (50.3%)	40 (39.2%)
Hemicryptophytes	H	368 (20.2%)	36 (21.8%)	26 (25.5%)
Chamaephytes	C	222 (12.2%)	24 (14.5%)	9 (8.8%)
Geophytes	G	169 (9.2%)	31(18.8%)	15 (14.7%)
Vines	V	78 (4.3%)	2 (1.2%)	1 (1%)
Phanerophyte shrubs	PhS	62 (3.4%)	1 (0.6%)	4 (3.9%)
Trees	T	54 (3.0%)	2 (1.2%)	4 (3.9%)
Parasites	P	27 (1.5%)	-	3 (2.9%)
Helophyte	HE	26 (1.4%)	-	2 (2.0%)
Biennials	F	1 (0.1%)	-	1 (1.0%)

**Table 7.** Distribution of chorotypes in the Palestinian flora. Species are categorised by their main chorotype.

Chorotype		Plant list (n = 1826)	Endemic plants (n = 165)	Threatened plants (n = 102)
Mediterranean	M	1094 (59.9%)	123 (74.5%)	48 (47.1%)
Irano-Turanian	IT	219 (12.0%)	17 (10.3%)	28 (27.5%)
Saharo-Arabian	SA	216 (11.8%)	24 (14.5%)	7 (6.9%)
Euro-Siberian	ES	144 (7.9%)	0 (0%)	7 (6.9%)
Tropical	T	53 (3.0%)	0 (0%)	4 (3.9%)
Pluri-regional-bor-trop	PT	40 (2.2%)	0 (0%)	1 (1%)
Sudanian	SUD	55 (3.0%)	1 (0.6%)	6 (5.9%)
American	A	4 (0.2%)	0 (0%)	0 (0%)
Others	Others	1 (0.1%)	0 (0%)	1 (1%)

**Table 8.** IUCN and the Conservation Measures Partnership (CMP) unified classification of direct threats* for SP red plants, showing the potential main causes threatening the existence of the plants.

**Threats**			**Number of Species**
**Level of Classification**			
1	2	3	
**1. Residential and commercial development**			
	1.1 Housing and Urban areas		8
	1.2 Commercial and industrial areas		3
	1.3 Tourism and recreation areas		5
**2. Agriculture and aquaculture**			
	2.1 Annual and perennial non-timber crops		
		2.1.1 Shifting agriculture	21
	2.3 Livestock farming and ranching		
		2.3.1 Nomadic grazing	5
**3. Energy production and mining**			
	3.2 Mining and quarrying		1
**4. Transportation and service corridors**			
	4.1 Roads and railroads		2
**5. Biological resource use**			
	5.2 Gathering terrestrial plants for intentional use		
		5.2.1 Intentional use (species being assessed is the target)	2
	5.3 Logging and wood harvesting		
		5.3.1 intentional use-small scale	1
**6. Human intrusions and disturbance**			
	6.1 Recreational activities		2
	6.2 Commercial and industrial areas		2
	6.3 Work and other activities		22
**7. Natural system modification**			
	7.1 Fire and fire suppression		1
		7.1.1 Increase in fire frequency/intensity	
		7.1.3 Fire and fire suppression-trend unknown/unrecorded	1
	7.2 Dams and water management use		
		7.2.1 Abstraction of surface water for domestic use	1
		7.2.5 Abstraction of groundwater for domestic use	4
**8. Invasive and other problematic species, genes and diseases**			0
**9. Pollution**			
	9.1 Domestic water and urban wastewater		
		9.1.1 Sewage	1
		9.1.3 Type unknown/unrecorded	2
	9.3 Agricultural and forestry effluent		
		9.3.3 Herbicides and pesticides	1
	9.4 Garbage and solid waste		1
**10. Geological events**			0
**11. Climate change and severe weather**			
	11.1 Habitat shifting and alteration		11
	11.2 Drought		5
**12. Other options**			
	12.1 **Other threats** (small population size)		54
**Total**			**156**

*IUCN (2019)
